# Supplementary material for: Structure and replication of Pseudomonas aeruginosa phage JBD30
Source: EMBO J. 2024 Aug 14;43(19):4384–405. doi: 10.1038/s44318-024-00195-1 (PMC11445458; doi:10.1038/s44318-024-00195-1)
Supplement: Supplementary file 1 — Appendix [file 44318_2024_195_MOESM1_ESM.pdf]

Supplementary Materials for

Structure and replication of Pseudomonas aeruginosa phage JBD30

Lucie Valentová[1], Tibor Füzik[1], Jiří Nováček[1], Zuzana Hlavenková[1], Jakub Pospíšil[1], and Pavel Plevka[1]

1 – Central European Institute of Technology, Masaryk University, Brno, Czech Republic

& – corresponding author: pavel.plevka@ceitec.muni.cz

This file includes:

Appendix Figure S1.	FSC curves of cryo-EM reconstructions.	2
Appendix Figure S2.	Local resolution maps of cryo-EM reconstructions.	4
Appendix Figure S3.	Characterization of protein composition of JBD30 virion.	5
Appendix Figure S4.	Packaging of dsDNA inside capsid of JBD30 virion.	6
Appendix Figure S5.	Electrostatic charge distribution of JBD30 connector and tail-forming protein oligomers.	7
Appendix Figure S6.	JBD30 baseplate and its comparison with that of bacteriophage T5.	8
Appendix Figure S7.	Translocation of phage JBD30 to *P. aeruginosa* cell surface is inhibited by low temperature.	9
Appendix Figure S8.	JBD30 tape measure protein *gp46*.	10
Appendix Figure S9.	One-step growth curve of phage JBD30 propagated on *P. aeruginosa* strain BAA-28.	11
Appendix Figure S10.	Expression of type IV pili by *P. aeruginosa* cells.	12
Appendix Figure S11.	Replication cycle of bacteriophage JBD30 visualized using lattice SIM fluorescence microscopy.	13
Appendix Figure S12.	Effect of DAPI labelling on phage JBD30 viability.	14
Appendix Figure S13.	Lattice SIM fluorescence microscopy of *P. aeruginosa* cells infected by DAPI-labelled JBD30.	15
Appendix Table S1.	Complete list of JBD30 encoded genes.	17
Appendix Table S2.	Data collection and structure quality indicators.	19
Appendix Table S3.	Homologues of selected JBD30 structural proteins.	20

Supplementary figures

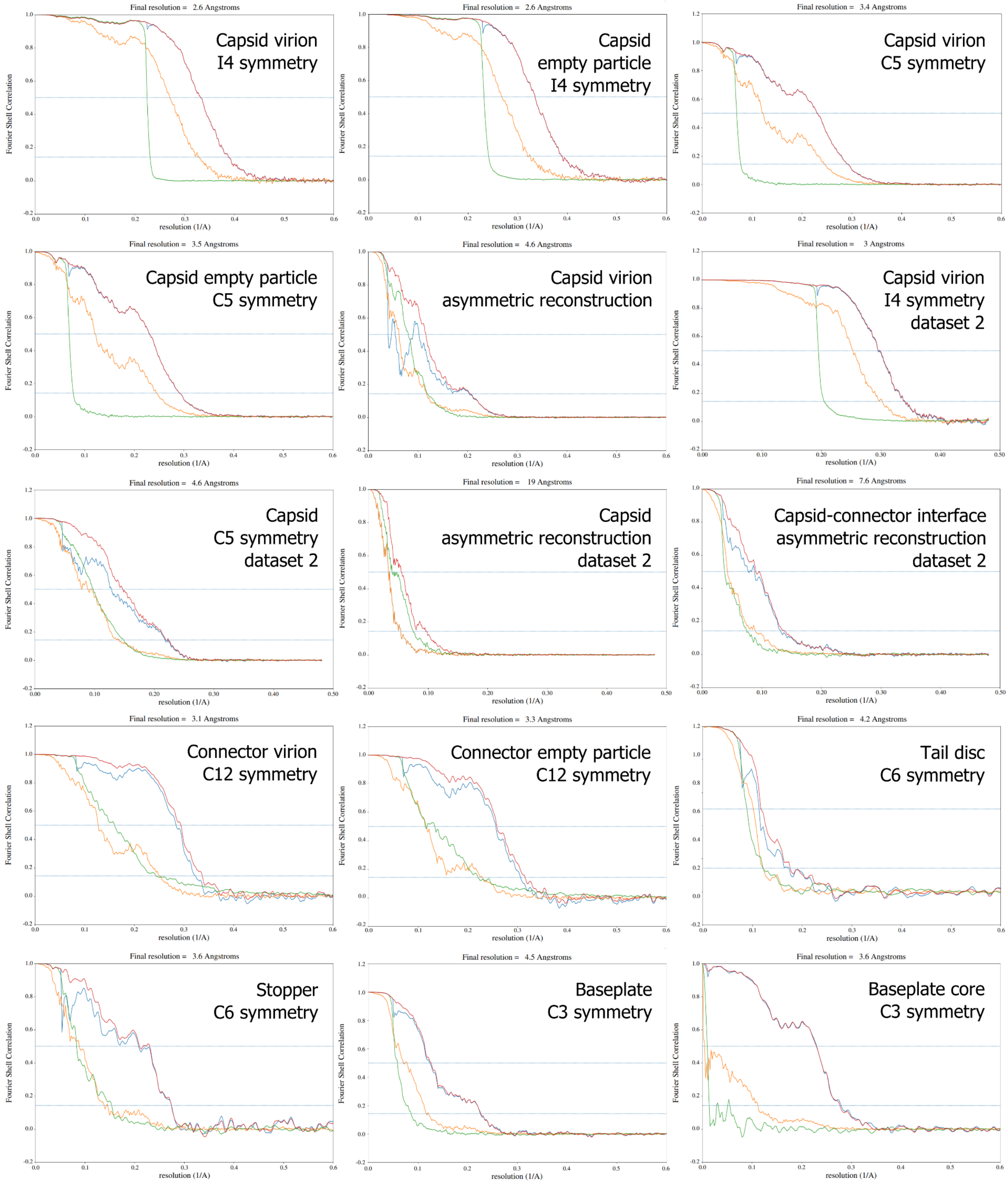

Appendix Figure S1. Continues on the next page.

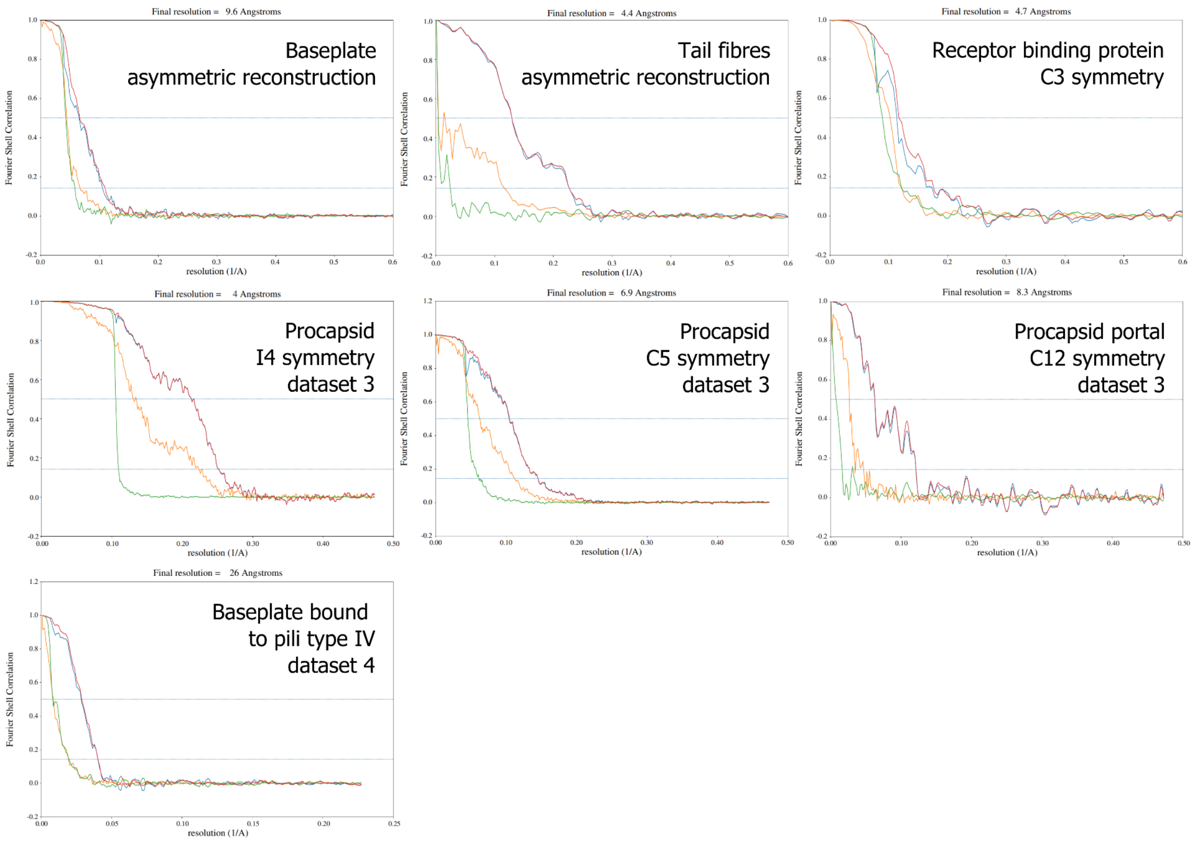

Appendix Figure S1. FSC curves of cryo-EM reconstructions. Fourier shell correlation curves of Fourier shell correlation corrected half-maps (blue), unmasked half-maps (orange), masked half-maps (red), and phase randomized masked half-maps (green) of individual cryo-EM reconstructions. The horizontal blue lines indicate the reference FSC values of 0.143 and 0.5. The final resolution is reported for the FSC cutoff at 0.143.

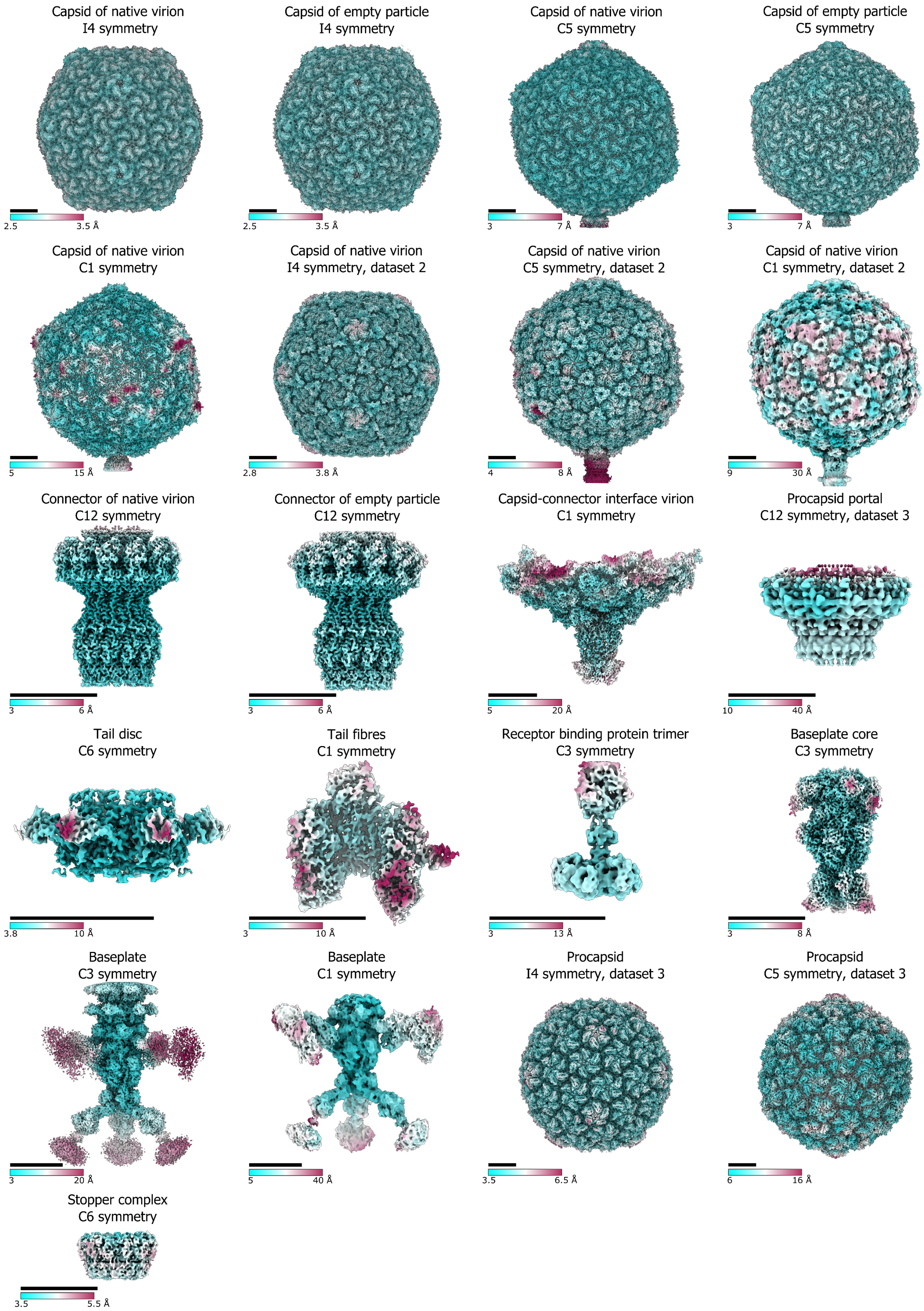

Appendix Figure S2. Local resolution maps of cryo-EM reconstructions. Local resolution estimation was calculated using RELION 3.1 (Zivanov *et al*, 2018; Kucukelbir *et al*, 2014). Scale bars correspond to 10 nm.

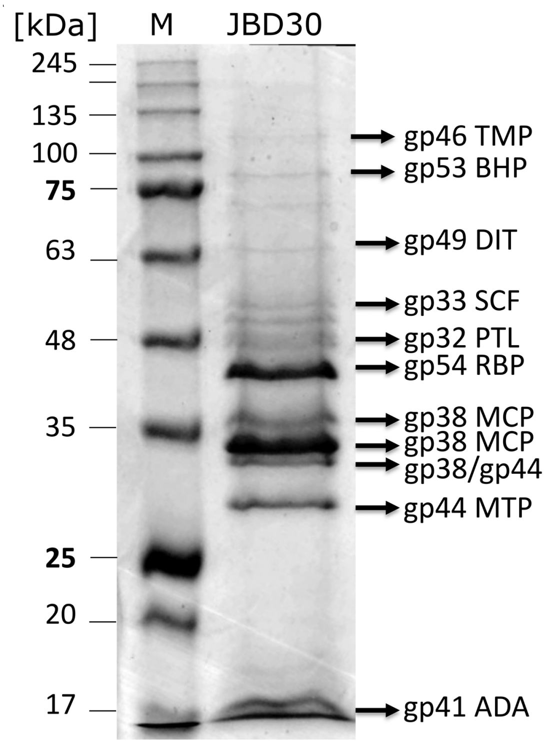

Appendix Figure S3. Characterization of protein composition of JBD30 virion. SDS-PAGE of proteins from bacteriophage JBD30. The types of proteins in bands were determined using MALDI MS/MS. The occurrence of one protein in multiple bands may be the result of protein fragmentation or incomplete separation in the gel. M – protein marker, JBD30 – purified phage sample, gp – gene product, TMP – tape measure protein, BHP – baseplate hub, DIT – distal tail protein, SCF – scaffolding protein, PTL – portal protein, RBP – receptor binding protein, MCP – major capsid protein, MTP – major tail protein, ADA – adaptor protein.

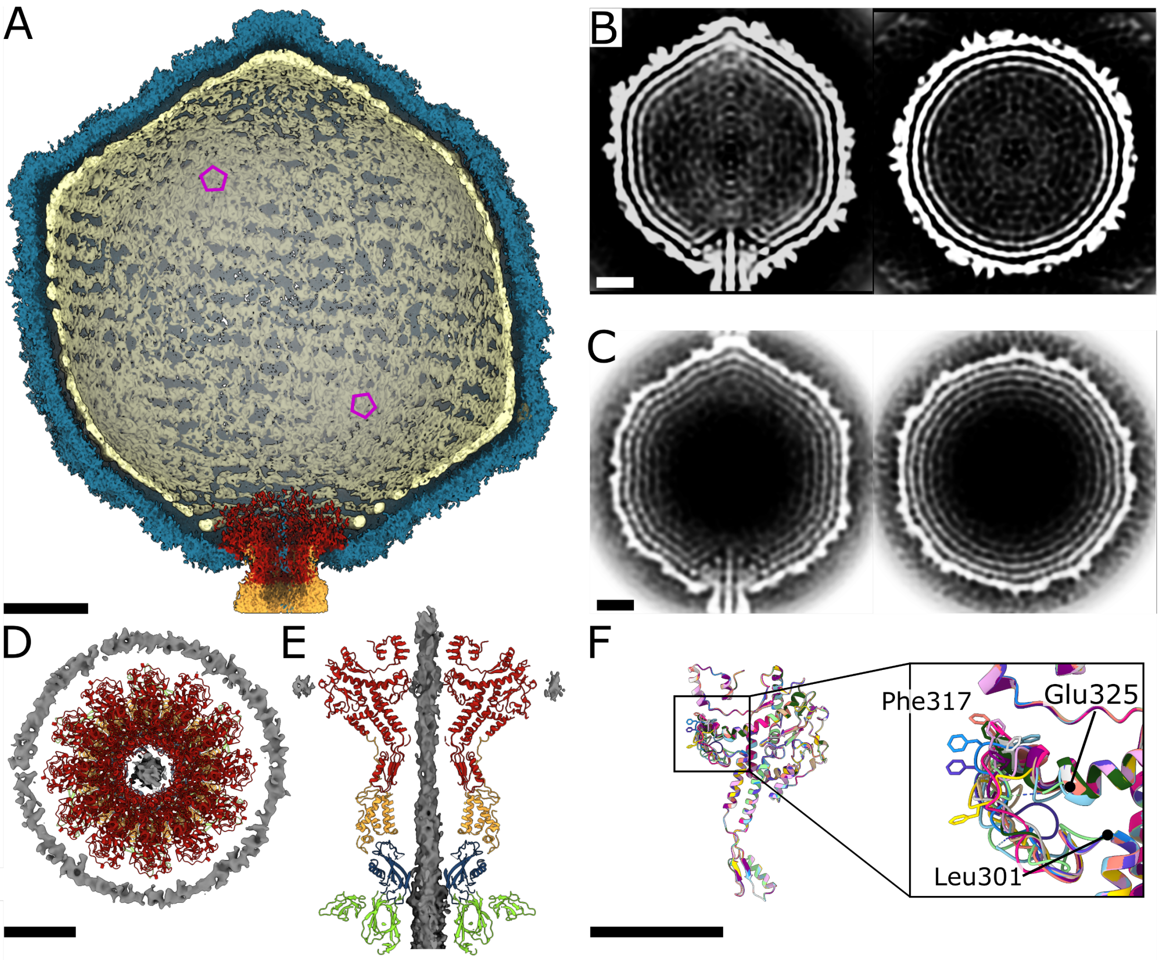

Appendix Figure S4. Packaging of dsDNA inside capsid of JBD30 virion. (A) Asymmetric reconstruction of JBD30 capsid (in blue) with front half removed, showing outermost layer of packaged dsDNA genome (yellow) lining the capsid wall. The ordering of the dsDNA is disrupted under fivefold vertices of the capsid. Density of the portal complex is shown in red, and that of the adaptor complex in orange. Positions of selected fivefold vertices are indicated by purple pentagons. Scale bar represents 10 nm. Central slices through fivefold symmetrized **(B)** and asymmetric **(C)** reconstructions of JBD30 head. The maps were low-pass filtered to 20 Å. Scale bars represent 10 nm. Top **(D)** and sideview **(E)** of interaction of packaged dsDNA with connector proteins. The proteins are shown in cartoon representation with portal proteins in red, adaptor proteins in orange, stopper proteins in blue, and major tail proteins in green. The density assigned to the dsDNA is highlighted in dark grey. Scale bar 5 nm. **(F)** Cartoon representation of the twelve portal proteins were superimposed to illustrate the differences in the Leu301–Glu325 loop.

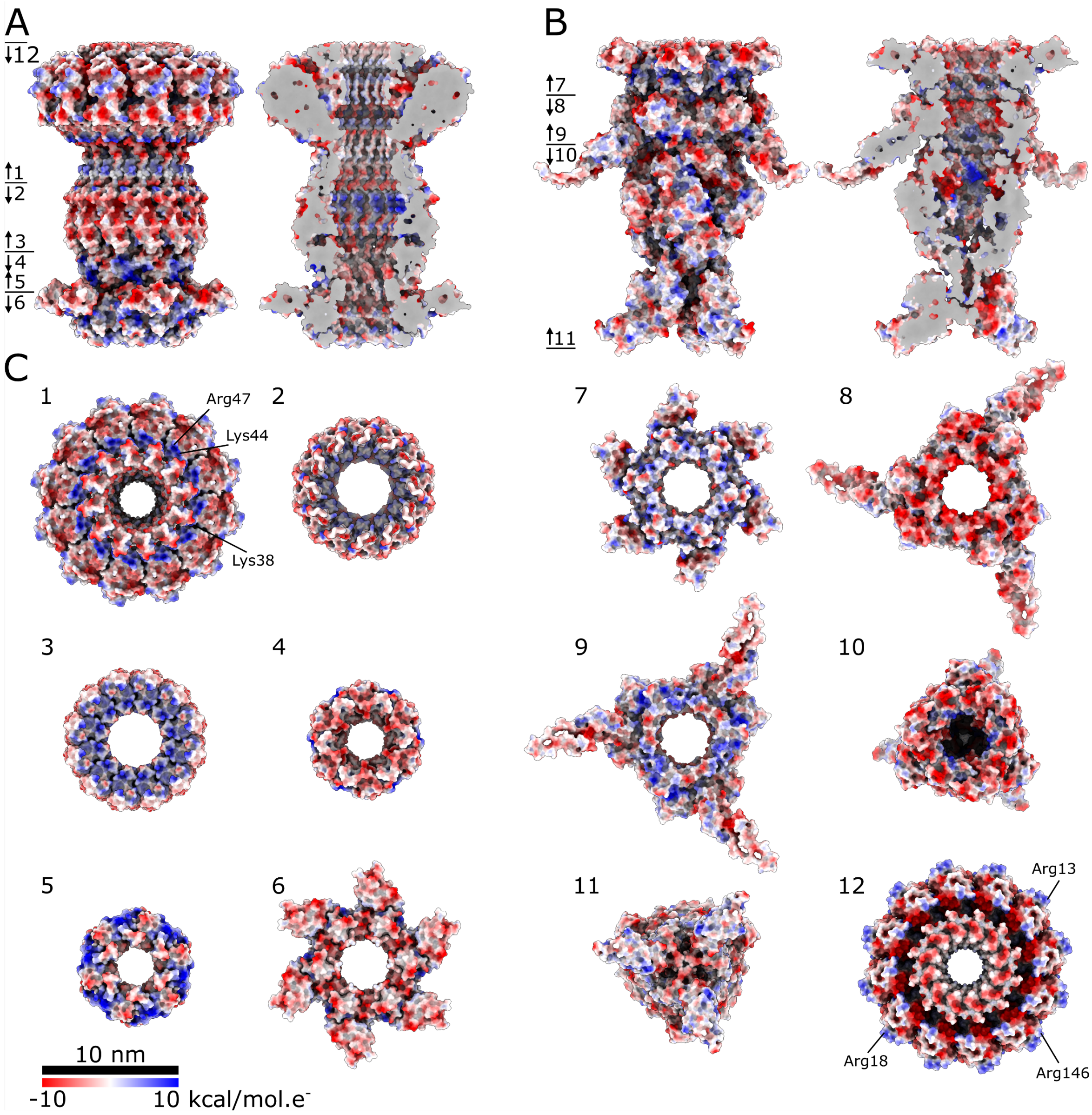

Appendix Figure S5. Electrostatic charge distribution of JBD30 connector and tail-forming protein oligomers. Molecular surface representation of **(A)** neck region and **(B)** baseplate core coloured according to electrostatic potential (±10 kcal/mol/e). Both the neck and the baseplate core are shown in a side view and then in a side view with the front half removed to show the electrostatic potential of the channel. **(C)** Top and bottom views of individual protein rings, the positions of the views are indicated by numbers and arrows in (A) and (B). 1 – portal protein dodecamer with highlighted residues interacting with the capsid, 2, 3 – adaptor protein dodecamer, 4, 5 – stopper protein hexamer, 6, 7 – major tail protein hexamer, 8, 9 – distal tail protein trimer, 10, 11 – baseplate hub protein trimer, 12 – portal protein dodecamer top view, residues interacting with the genome are highlighted.

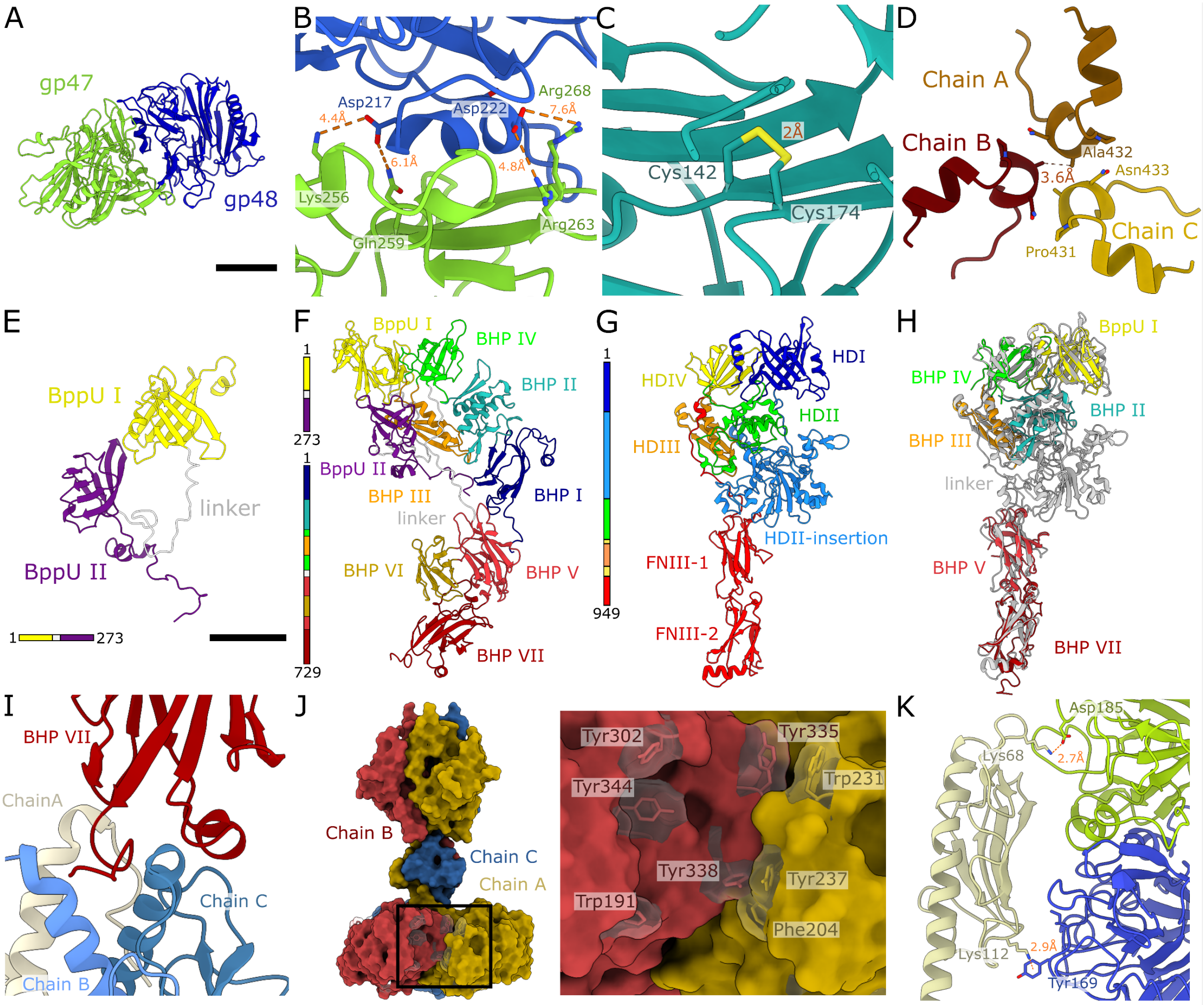

Appendix Figure S6. JBD30 baseplate and its comparison with that of bacteriophage T5. (A) Cartoon representation of tail fibre forming proteins *gp47* (green) and *gp48* (blue). **(B)** Interaction of helix Pro216–Ser224 of JBD30 *gp48* (ultramarine blue) with *gp47* (green). The proteins are shown in cartoon representation and the side chains of the interacting residues are shown in stick representation. Distances between selected atoms are indicated. **(C)** Disulphide bond between Cys142 and Cys174 of tail fibre protein *gp48*. **(D)** Cartoon representation of baseplate tip formed by three baseplate hub proteins as seen from baseplate interior. **(E)** Cartoon representation of upper baseplate protein (BppU) with domains coloured according to the sequence diagram at the bottom of the panel. **(F)** Cartoon representation of JBD30 baseplate hub protein (BHP), upper baseplate protein complex coloured according to the sequence diagrams on the left of the panel. **(G)** Cartoon representation of bacteriophage T5 baseplate hub protein pb3 coloured according to domains (Linares *et al*, 2022) (HD – Hub domain, FN – Fibronectin domain). **(H)** Superposition of JBD30 baseplate hub protein and baseplate upper protein domains coloured as in (F) onto T5 phage baseplate hub protein shown in grey. **(I)** Interactions between hub protein and trimer of receptor binding proteins. The C-terminus of the hub protein (red) is wedged into the pockets formed by N-terminal thigh domains of the three receptor binding proteins **(J)** Molecular surface representation of trimer of receptor binding proteins, and detail of putative O-antigen binding cleft between two receptor binding protein foot domains rich in aromatic residues. **(K)** Cartoon representation of the interactions between *P. aeruginosa pilA* (beige) and JBD30 tail fibre forming heterodimer of *gp47* (green) and *gp48* (ultramarine blue) predicted using Alphafold2 multimer (Evans *et al*, 2021). The side chains of the interacting residues are shown in stick representation. Distances between selected atoms are indicated.

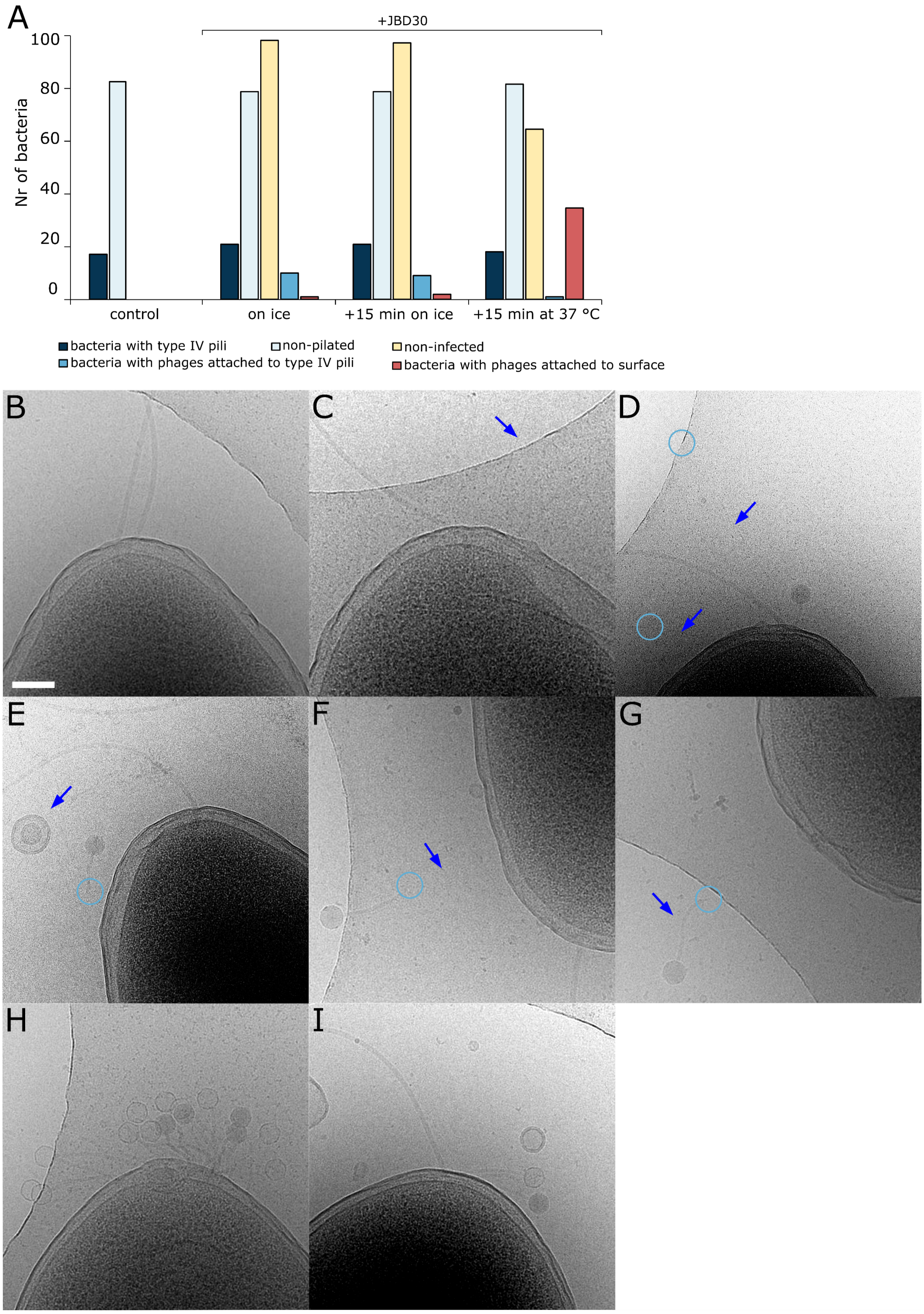

Appendix Figure S7. Translocation of phage JBD30 to *P. aeruginosa* cell surface is inhibited by low temperature. **(A)** Plot of *P. aeruginosa* cells forming type IV pili and distribution of JBD30 particles on infected *P. aeruginosa* cells. One hundred cells were analyzed for each group. Images of cells from each of the analyzed groups: **(B)** control, **(C)** cells incubated with JBD30 for 15 min on ice, and **(D)** cells incubated with JBD30 for 15 min on ice and for additional 15 min at 37°C, **(E)** cells incubated with JBD30 for 30 min on ice. Blue arrows point to type IV pili, contacts between the JBD30 baseplates and type IV pili are highlighted with cyan circles. Scale bar represents 100 nm.

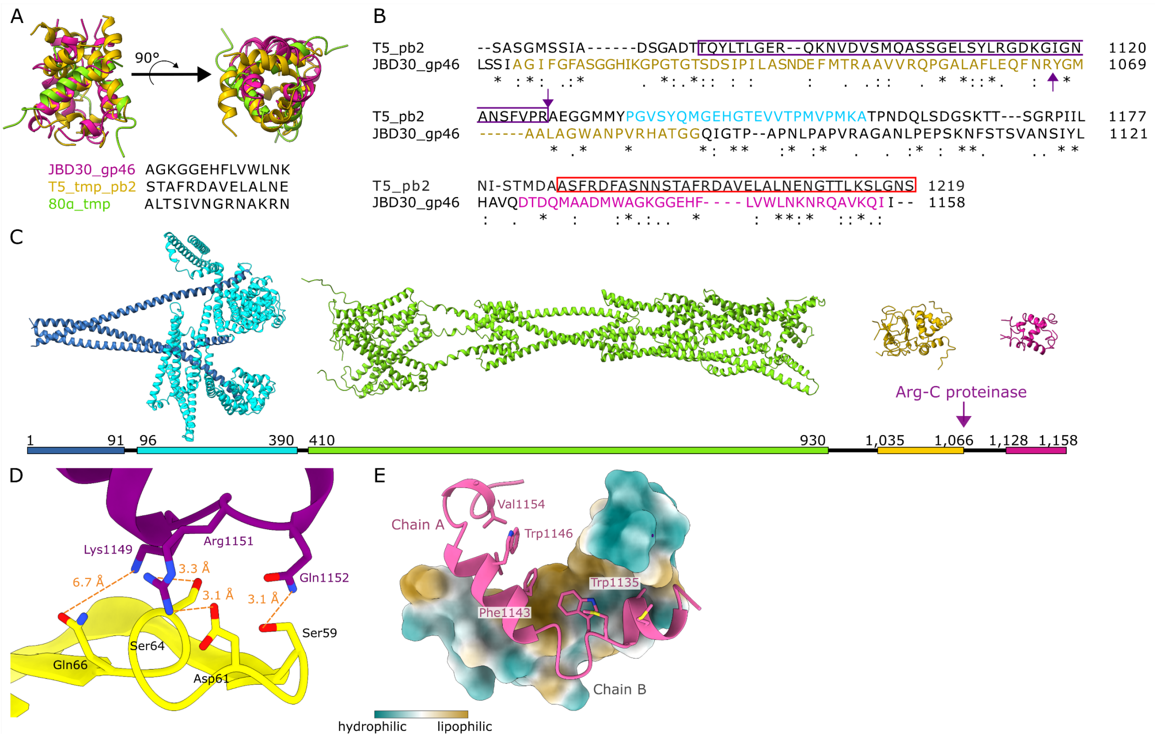

Appendix Figure S8. JBD30 tape measure protein *gp46*. (A) Cartoon representation of superposition of C-terminus (residues 1,128–1,158) of JBD30 tape measure protein (magenta) with corresponding parts of tape measure proteins of phages T5 (gold) and 80α (green). A structure-based sequence alignment is shown at the bottom of the panel. **(B)** Sequence alignment of JBD30 tape measure protein and phage T5 tape measure protein pb2. The JBD30 sequence is coloured according to domains: the C-terminus, which is resolved in the JBD30 structure, is shown in magenta, the hook-forming domain in gold, the purple arrow points to the predicted Arg-C cleavage site. The sequence of the T5 the tape measure protein is marked: the zinc carboxypeptidase motif is shown in blue, the red box indicates parts of the protein resolved in the structure of the T5 virion, the purple box indicates the part resolved in baseplates bound to the FhuA-nanodiscs (Linares *et al*, 2022). **(C)** Cartoon representation of structure of JBD30 tape measure protein trimer predicted using Alphafold2 multimer (Evans *et al*, 2021). Individual domains are coloured according to the sequence diagram shown at the bottom of the panel: blue – N-terminus, cyan – N-terminal α-helical bundles, green – region forming putative channel for DNA translocation across bacterial membranes, gold – hook-forming domain, magenta – resolved C-terminal domain. The purple arrow points to the predicted Arg-C cleavage site. **(D)** Cartoon representation of tape measure protein C-terminus (purple) interacting with upper baseplate protein domain I (yellow). Distances between selected atoms are indicated. **(E)** Hydrophobic core of trimer formed by C-terminal domains of tape measure proteins. Only two chains from the trimer are shown. One chain is depicted in cartoon representation, and the other chain is shown as a molecular surface coloured according to hydrophobicity. Hydrophobic side chains of the first chain pointing towards the centre of the trimer are shown in stick representation.

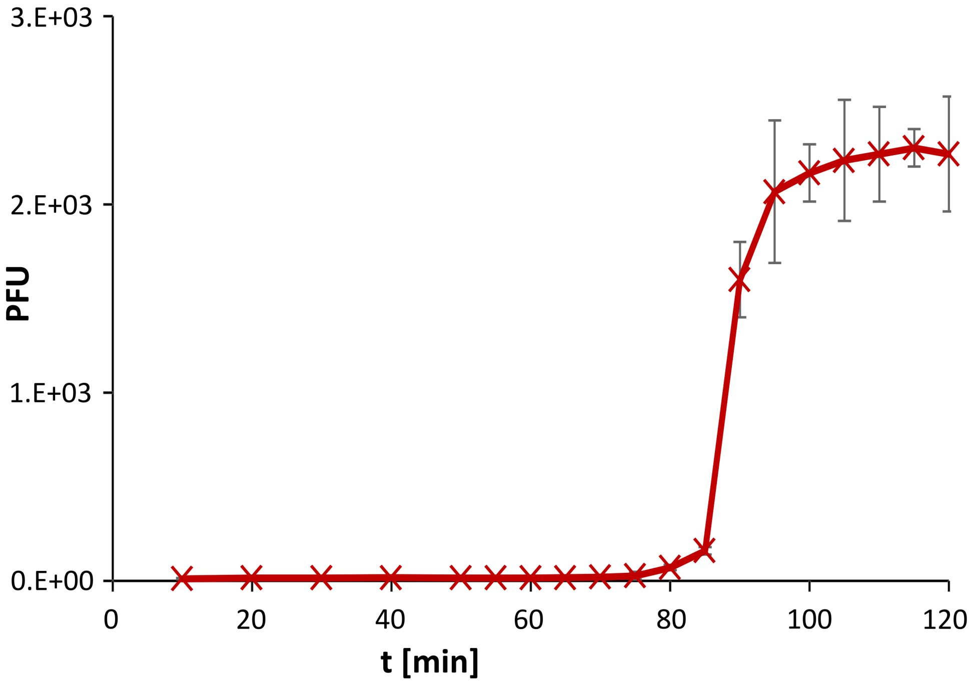

Appendix Figure S9. One-step growth curve of phage JBD30 propagated on *P. aeruginosa* strain BAA-28. The latent period of JBD30 infection lasts approximately 85 min. Average burst size is 140 ± 20 virions per infected cell. The curve is an average of three independent experiments. The error bars show standard deviations.

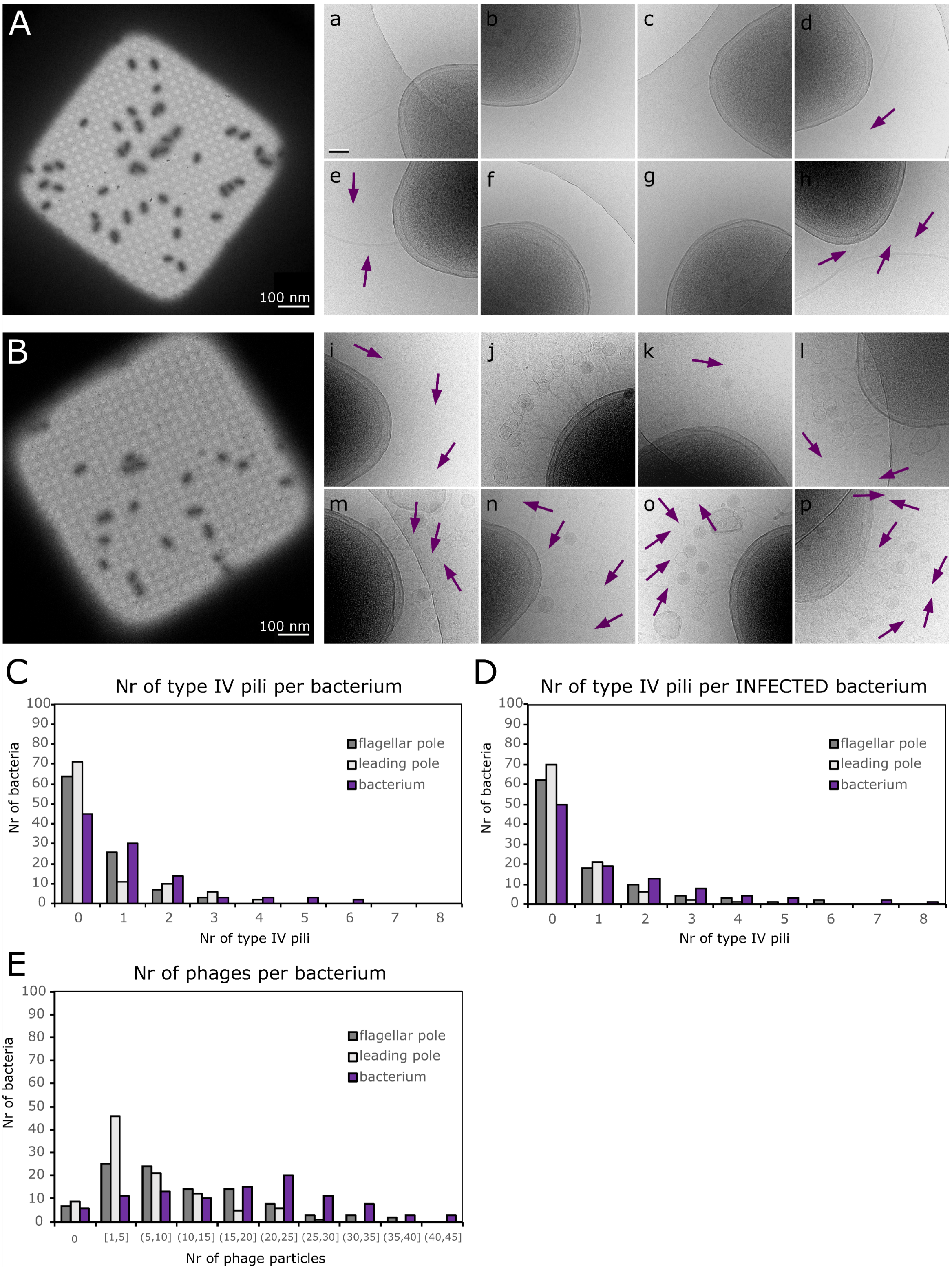

Appendix Figure S10. Expression of type IV pili by *P. aeruginosa* cells. (A) Control non-infected cells of *P. aeruginosa* imaged using cryo-EM, the pairs of panels **ab, cd, ef,** and **gh** show opposite poles of four selected cells. Purple arrows point to type IV pili. **(B)** *P. aeruginosa* cells infected with JBD30 virions at MOI 500 imaged three min post-infection. Images **i–p** show cell poles of 8 selected cells, purple arrows point to type IV pili. **(C)** In total 100 control and **(D)** 100 infected *P. aeruginosa* cells were imaged to calculate the average number of type IV pili formed by individual cells. **(E)** Distribution of phage particles attached to 100 infected *P. aeruginosa* cells.

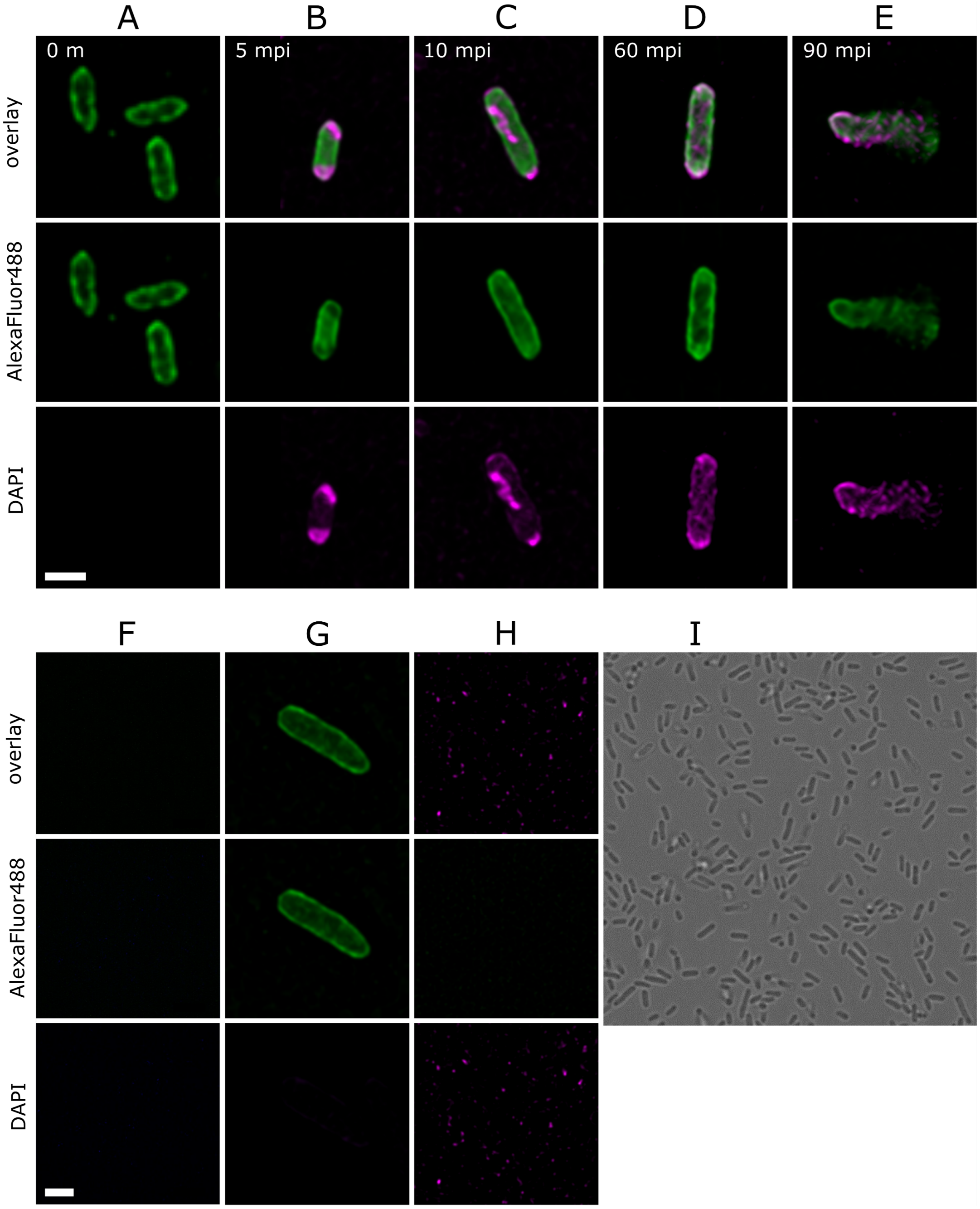

Appendix Figure S11. Replication cycle of bacteriophage JBD30 visualized using lattice SIM fluorescence microscopy. The phage genome is labelled with DAPI (magenta), *P. aeruginosa PAO1 pilA-A86C* cells are labelled with Alexa Fluor 488 C5 maleimide (green). Scale bars 1 µm, unless indicated otherwise. Mpi - min post-infection. **(A)** Non-infected cells of *P. aeruginosa*. **(B)** *P. aeruginosa* cells infected with JBD30. The phage signal is localized to the cell poles, since the phages utilize type IV pili to attach to cells. **(C)** Delivery of JBD30 genome into host cell. **(D)** Assembly of new virions. The DAPI signal becomes granular as the labelled DNA is filled into the capsids. **(E)** Cell lysis and release of phage progeny. **(F)** Control sample showing non-labelled cells of *P. aeruginosa.* Scale bar 1 µm. No fluorescence signal is observed. **(G)** Mock infected *P. aeruginosa* cells labelled with C5 maleimide. The absence of any DAPI signal indicates that there is no free DAPI in the phage lysate. **(H)** JBD30 virions labelled with DAPI. **(I)** *P. aeruginosa* cells imaged in transmitted light. Scale bar 5 µm. Please note that images shown in panels A-E are identical to those displayed in Fig. 7 F,H, L, J.

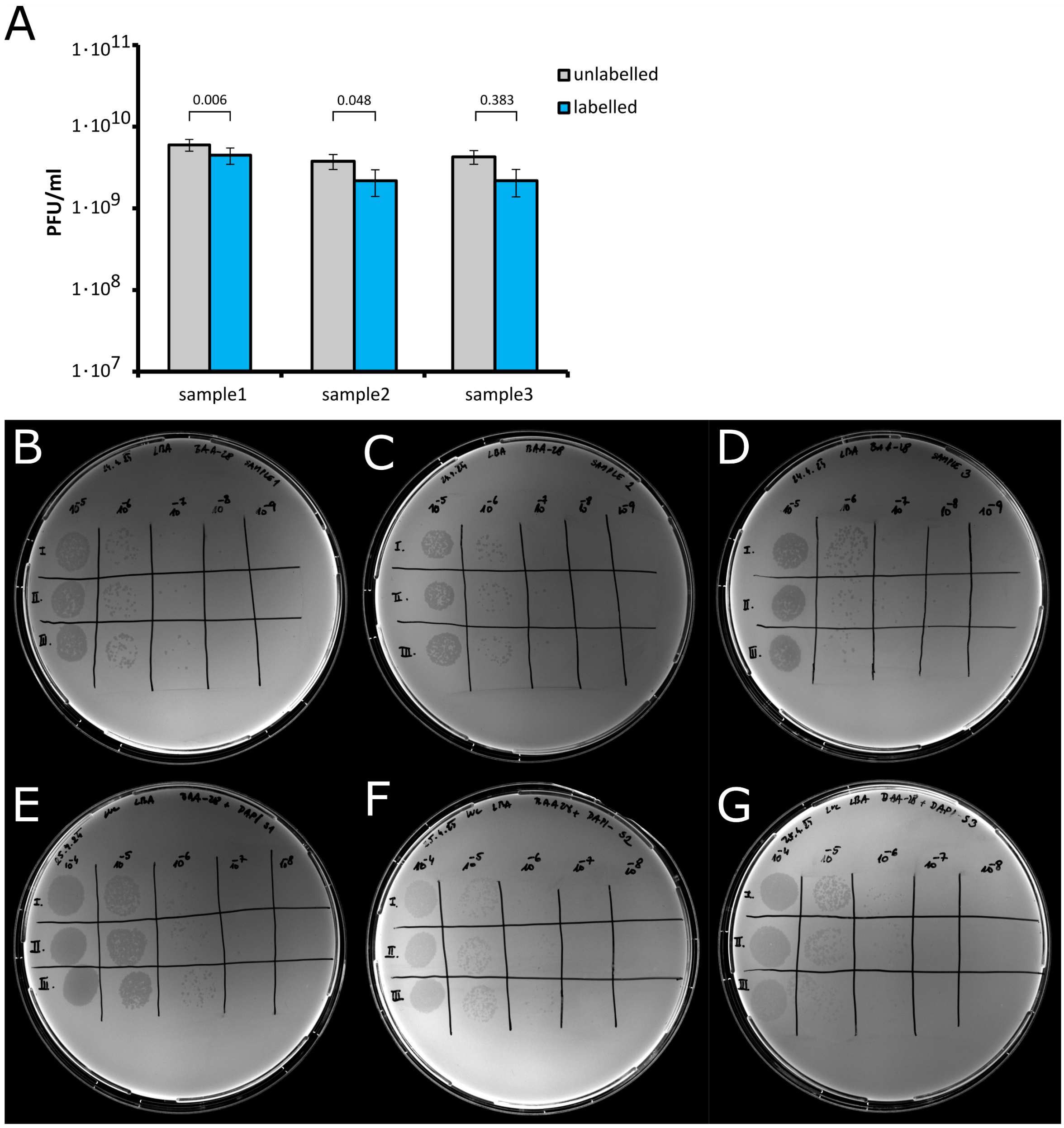

Appendix Figure S12. Effect of DAPI labelling on phage JBD30 viability. (A) Titer of JBD30 lysate (PFU/ml) was measured before and after labelling with DAPI. Phage titer was measured in triplicates in three independent biological experiments. The error bars represent standard deviations. The effect of DAPI staining of JBD30 viability is limited. **(B-G)** Lawns of *P. aeruginosa* BAA-28 used for determining the titer of unlabelled and DAPI-labelled JBD30.

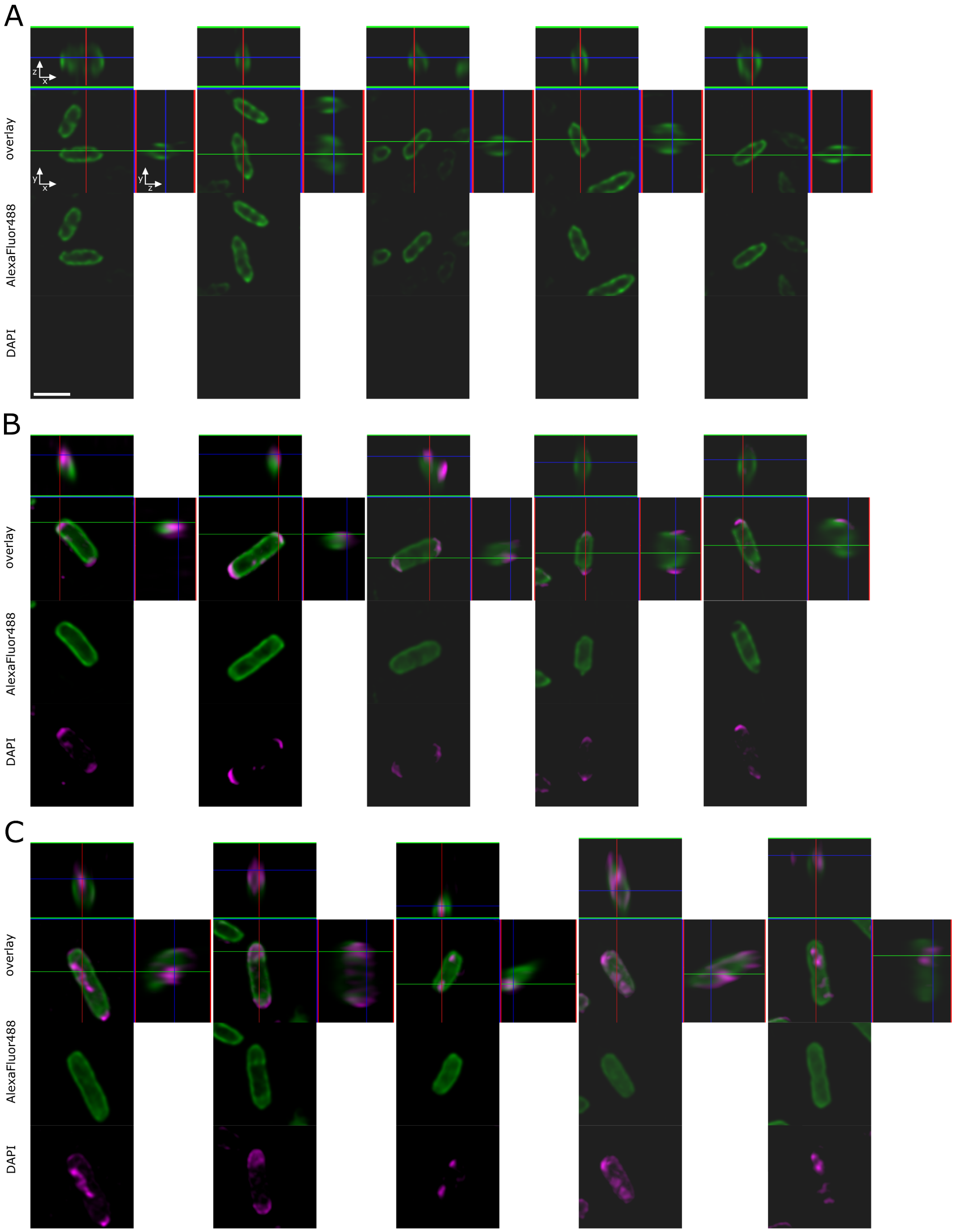

Appendix Figure S13. Lattice SIM fluorescence microscopy of *P. aeruginosa* cells infected by DAPI-labelled JBD30. Continues on the next page.

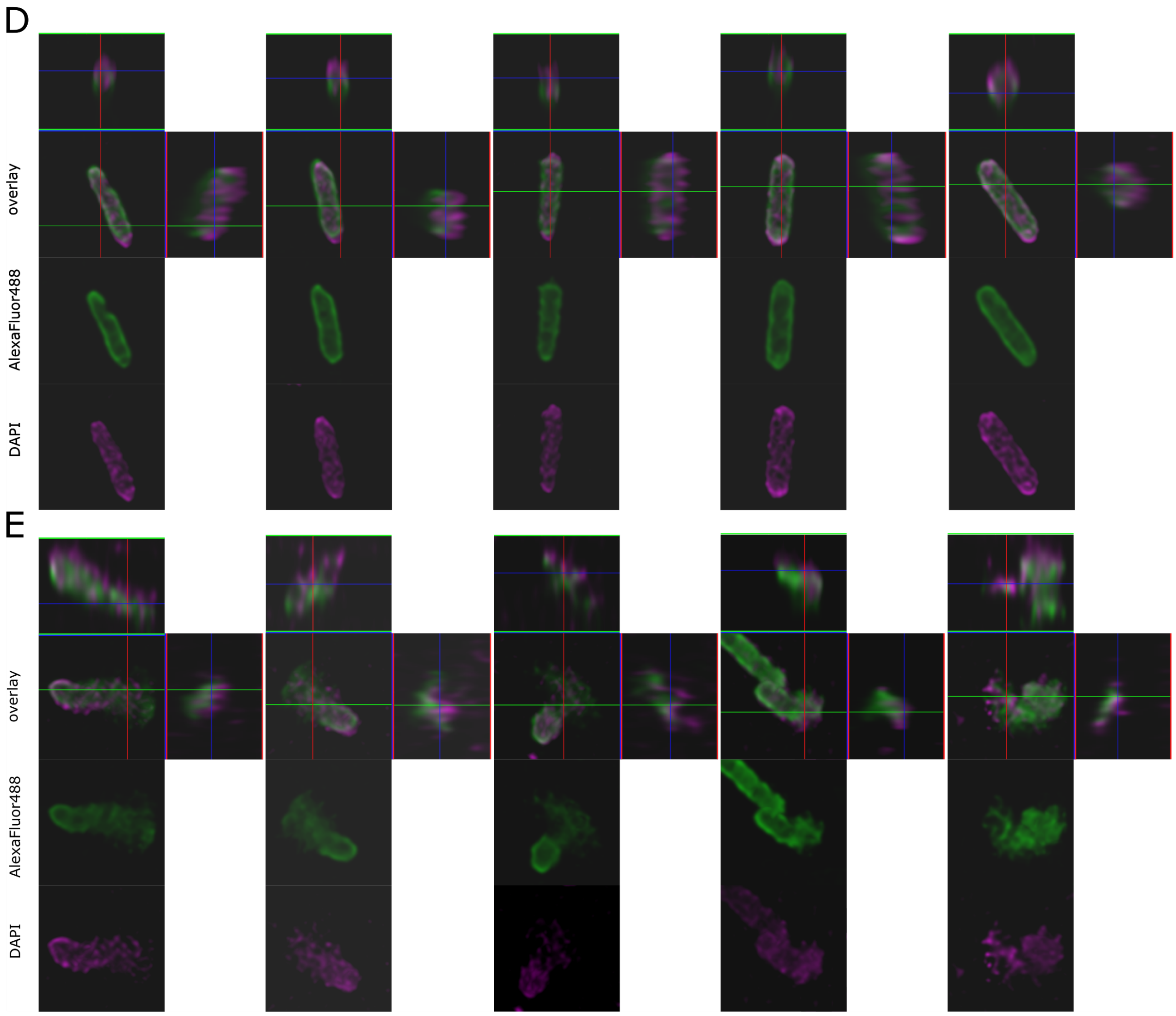

Appendix Figure S13. Lattice SIM fluorescence microscopy of *P. aeruginosa* cells infected by DAPI-labelled JBD30. xz and yz sections through z-stacks of IM images. The phage genome is labelled with DAPI (magenta), *P. aeruginosa* cells are labelled with Alexa Fluor 488 C5 maleimide (green). mpi - min post-infection, scale bar 1 µm. **(A)** Non-infected *P. aeruginosa* cells. **(B)** *P. aeruginosa* cells infected by JBD30 at 5 mpi. **(C)** Delivery of JBD30 genome into host cell, 10 mpi **(D)** Assembly of new virions, 60 mpi. **(E)** Cell lysis and release of phage progeny, 90 mpi.

Appendix Table S1. Complete list of JBD30 encoded genes. Genes encoding structural proteins are highlighted in bold. Homologues were searched for using BLAST, DALI (Holm, 2022), HHPred (Zimmermann *et al*, 2018), Pfam (Mistry *et al*, 2021), IterPro (Paysan-Lafosse *et al*, 2023) and Foldseek (van Kempen *et al*, 2023). Continues on the next page.

Orf	Gene product	Structural/ Found in MS analysis	No. of residues	Symmetry	No. of chains in virion	Modelled residues	Homologue proteins
1	Repressor cI	no/no	219	-	-	-	phage MP22 ORF1, *E. coli* phage 186 repressor c1, λ repressor c1 3BDN
2	Ner like transcription regulator	no/no	118	-	-	-	phage DMS3 *Ner*
3	Repressor cII	no/no	144	-	-	-	phage P22 CII repressor 1ADRa
4	Moron gene	no/no	108	-	-	-	affects bacterial cell twitching motility (Tsao *et al*, 2018)
5	Aqs1 protein	no/no	84	-	-	-	quorum sensing anti-activator, phage JBD24_4 Aqs1, phage DMS3 Aqs1, JBD30 Aqs1 (Bondy-Denomy *et al*, 2015)
6	Transposase A	no/no	689	-	-	-	Enterobacteria phage Mu Transposase 4FCY
7	Transposase B	no/no	250	-	-	-	Enterobacteria phage Mu Transposase 4FCY
8	Hypothetical protein	no/no	221	-	-	-	-
9	Moron gene	no/no	211	-	-	-	affects bacterial surface LPS O-antigen production (Tsao *et al*, 2018)
10	Hypothetical protein	no/no	38	-	-	-	-
11	Hypothetical protein	no/no	137	-	-	-	-
12	Hypothetical protein	no/no	97	-	-	-	-
13	Host-nuclease inhibitor Gam family protein	no/no	172	-	-	-	host-nuclease inhibitor Gam family protein of *Pseudomonas aeruginosa*
14	Moron gene	no/no	67	-	-	-	affects bacterial growth rate (Tsao *et al*, 2018)
15	Hypothetical protein	no/no	102	-	-	-	-
16	Hypothetical protein	no/no	93	-	-	-	-
17	Hypothetical protein	no/no	68	-	-	-	-
18	Hypothetical protein	no/no	89	-	-	-	-
19	Hypothetical protein	no/no	98	-	-	-	-
20	Regulatory protein GemA	no/no	135	-	-	-	multispecies: regulatory protein GemA
21	Middle operon regulator	no/no	149	-	-	-	Middle operon regulator Mor phage Mu, 1RR7
22	Hypothetical protein	no/no	115	-	-	-	-
23	Hypothetical protein	no/no	140	-	-	-	-
24	Hypothetical protein	no/no	138	-	-	-	-
25	Hypothetical protein	no/no	39	-	-	-	-
26	Hypothetical protein	no/no	133	-	-	-	-
27	Endonuclease	no/no	37	-	-	-	phage T4 endonuclease 2END
28	Hypothetical protein	no/no	62	-	-	-	-
29	DUF1804 family protein	no/no	166	-	-	-	DUF1804 family protein *Pseudomonas aeruginosa*
30	Moron gene	no/no	164	-	-	-	blocks phage genome injection (Tsao *et al*, 2018)
31	Terminase large subunit	no/no	550	-	-	-	phage T4 large terminase 3CPE, phage D6E large terminase subunit: 5OE8, phage Sf6 terminase 4IDH

Appendix Table S1. Complete list of JBD30 encoded genes - Continued.

32	**Portal protein**	yes/yes	526	C12	12	5–409	phage G20C portal protein 4ZJN, phage T4 portal protein gp20 3JA7, HK97 family phage portal protein 3KDR-C, phage SPP1 portal protein 2JES
33	Scaffolding protein	yes/yes	428	?	in procapsid	-	Phage Mu protein F like protein VVH83508.1
34	Virion morphogenesis protein	no/no	155	-	-	-	virion morphogenesis protein of *Pseudomonas aeruginosa*
35	Anti-CRISPR protein Acrf1	no/no	78	-	-	-	JBD30 AcrF1 protein 5XLO
36	Anti-CRISPR associated regulatory protein Aca1	no/no	79	-	-	-	JBD30 Aca1 anti-CRISPR-associated protein 7C0B
37	Prohead protease	no/no	365	-	-	-	phage protease *P. aeruginosa* seq. ID: WP_003137267.1
38	**Major capsid protein**	yes/yes	304	I4	415	2–151, 160–304	GTA major capsid protein 6TSW, phage HK97 major capsid protein 1OHG, phage T7 major capsid protein 10A 3J7W
39	**Minor capsid protein**	yes/no	113	C3	420	53–108	Phage T4 Soc protein 5VF3
40	Hypothetical protein	no/no	55	-	-	-	-
41	**Adaptor protein**	yes/yes	138	C12	12	2–138	phage MU neck protein J 5YDN, phage HK97 gp6 3JVO, GTA adaptor 6TOA
42	**Stopper protein**	yes/no	157	C6	6	9–32, 36–147	phage λ tail tube terminator gpU 3FZ2, phage SPP1 head-to-tail protein 2LFP, GTA adaptor protein 6TE9
43	DNA packaging chaperone	no/no	59	-	-	-	phage λ DNA-packaging protein FI, chaperone 2LSM
44	**Major tail protein**	yes/yes	256	C6	270	5–256	Phage YSD1_22 major tail protein 6XGR, SPP1 tail tube protein gp17.1* 6YEG
45	Tail assembly chaperone	no/no	164	-	-	-	phage HK97 tail assembly chaperone 2OB9, phage TAC13 tail assembly chaperone
46	**Tape measure protein**	yes/yes	1158	C3	3	730–761	phage P22 tape measure trimeric coiled-coil protein 4LIN, phage HK97 tape measure protein, phage λ tail tape measure protein
47	**Tail fibre hydrolase**	yes/no	318	C1	9	2–318	cl11424: residues 274 - 315 nitrilase superfamily protein, including nitrile- or amide-hydrolysing enzymes and amide-condensing enzymes
48	**Tail fibre**	yes/no	307	C1	9	2–306	-
49	**Distal tail protein**	yes/yes	567	C3	3	14–567	phage T5 distal tail protein 6F2M
50	**Upper baseplate protein**	yes/no	273	C3	3	2–273	GTA baseplate 6TEH, phage PVC baseplate 6J0M, phage MUSO2 tail protein 3CDD, phage P2 putative receptor binding protein 2WZP
51	Hypothetical protein	no/no	76	-	-	-	-
52	Hypothetical protein	no/no	72	-	-	-	-
53	**Baseplate hub**	yes/yes	735	C3	3	2–729	Phage T5 baseplate hub protein 7Zqp, GTA Megatron protein 6TEH, T4 phage tail-associated lysozyme 2Z6B
54	**Receptor binding protein**	yes/yes	382	C3	9	10–382	Phage vB_ApiP_P1 tail spike 6E1R, phage CBA120 6NW9, endo-1,4-beta-xylanase C 4XUP, arabinogalactan endo-1,4-beta-galactosidase 2XOM, endoglucanase 2C26
55	Hypothetical protein	no/no	95	-	-	-	-
56	Hypothetical protein	no/no	74	-	-	-	-

Appendix Table S2. Data collection and structure quality indicators. In addition, baseplate composite map and structure (EMDB-19256, PDB-8RK3) and capsid-neck composite map and structure (EMDB-19439, PDB-8RQE) have been deposited.

	**Capsid virion**	**Capsid empty particle**	**Capsid virion**	**Capsid empty particle**	**Capsid virion**	**Capsid virion with mCP**	**Capsid virion with mCP**	**Capsid virion with mCP**	**Capsid – connector mCP**	**Connector virion**	**Connector empty particle**
Dataset number	2	2	2	2	2	1	1	1	1	2	2
Detector	K3	K3	K3	K3	K3	K2	K2	K2	K2	K3	K3
Magnification	105 000	105 000	105 000	105 000	105 000	130 000	130 000	130 000	130 000	105 000	105 000
Voltage (kV)	300	300	300	300	300	300	300	300	300	300	300
Exposure $(e^{-}\cdot A^{-2})$	34	34	34	34	34	30	30	30	30	34	34
Pixel size (Å)	0.8336	0.8336	0.8336	0.8336	0.8336	1.04	1.04	1.04	1.04	0.8336	0.8336
Symmetry	I4	I4	C5	C5	C1	I4	C5	C1	C1	C12	C12
Initial number of particles	5 167	3 037	5 167	3 037	3 567	13 129	5 360	2 913	5 360	4 713	2 666
Final number of particles	4 713	2 666	3 567	1 878	1 990	5 360	2 913	1 364	2 913	2 076	1 142
Initial model	*de novo*	*de novo*	I4 map of virion capsid	I4 map of empty capsid	C5 map of virion capsid	*de novo*	I4 map of virion capsid with mCP	C5 map of virion capsid with mCP	I4 map of virion capsid with mCP	C5 map of virion capsid	C5 map of empty particle capsid
Map resolution (Å)	2.57	2.58	3.37	3.52	4.93	2.95	4.60	18.59	7.61	3.12	3.28
FSC threshold	0.143	0.143	0.143	0.143	0.143	0.143	0.143	0.143	0.143	0.143	0.143
B-factor for map sharpening	34.6	5.8	-45.0	0.0	-80.0	-83.8	-81.0	-80	-77.3	-64.8	-56.0
**EMDB**	19280	19281	19274	19273	19272	19270	19271	19269	19268	19267	19266
**PDB**	8RKN	8RKO	/	/	/	8RKC	/	/	/	8RKB	8RKA
**EMPIAR**	12062	12062	12062	12062	12062	12061	12061	12061	12061	12062	12062

	**Stopper**	**Tail**	**Baseplate**	**Baseplate core**	**Baseplate**	**Tail fibres**	**Receptor binding protein**	**Procapsid**	**Procapsid**	**Procapsid portal**	**Baseplate attached to type IV pilus**
Dataset number	2	2	2	2	2	2	2	3	3	3	4
Detector	K3	K3	K3	K3	K3	K3	K3	K2	K2	K2	K3
Magnification	105 000	105 000	105 000	105 000	105 000	105 000	105 000	130 000	130 000	130 000	42 000
Voltage (kV)	300	300	300	300	300	300	300	300	300	300	300
Exposure $(e^{-}\cdot A^{-2})$	34	34	34	34	34	34	34	30	30	30	22.4
Pixel size (Å)	0.8336	0.8336	0.8336	0.8336	0.8336	0.8336	0.8336	1.057	1.057	1.057	2.2
Symmetry	C6	C6	C3	C3	C1	C1	C3	I4	C5	C12	C1
Initial number of particles	3 570	1 780	8 376	8 376	8 376	5 337	5 340	783	783	404	703
Final number of particles	3 559	1 759	1 780	1 780	1 780	3 927	4 088	404	426	157	603
Initial model	*C6 map of connector*	C3 map of baseplate	*de novo*	C3 map of baseplate	C3 map of baseplate	C3 map of baseplate	C3 map of baseplate	*de novo*	*de novo*	C5 map of procapsid	C1 map of baseplate
Map resolution (Å)	3.65	3.88	4.46	3.60	9.00	4.54	4.74	4.01	6.92	8.33	26
FSC threshold	0.143	0.143	0.143	0.143	0.143	0.143	0.143	0.143	0.143	0.143	0.143
B-factor for map sharpening	-71.2	-111.9	-74.9	-72.6	0	-80	-90	-4.9	-108.5	0	0
**EMDB**	19265	19264	19263	19262	19261	19260	19259	19285	19258	19257	19561
**PDB**	8RK9	8RK8	8RK7	8RK6	/	8RK5	8RK4	8RKX	/	/	/
**EMPIAR**	12062	12062	12062	12062	12062	12062	12062	12063	12063	12063	12064

Appendix Table S3. Homologues of selected JBD30 structural proteins.

JBD30 protein		No. of residues available for alignment / No. of residues in protein	protein homologue	No. of residues available for alignment / No. of residues in protein	RMSD [Å]	% of residues used in alignment
gp38	hexamer forming major capsid protein	303/304	pentamer forming major capsid protein	303/304	0.659	89
gp42	stopper protein	139/157	*Rhodobacter capsulatus* GTA PDB-6te9-F	112/112	2.840	22
gp42	stopper protein	139/157	bacteriophage SPP1 *gp17* PDB-2lfp	139/139	3.307	27
gp42	stopper protein	139/157	bacteriophage λ *gpU* PDB-3fz2	134/134	2.101	12
gp46	tape measure protein C-terminus	32/1,158	bacteriophage T5 PDB-7zqb	35/1,219	1.067	48
gp46	tape measure protein C-terminus	32/1,158	bacteriophage 80α PDB-6v8i	20/1,154	0.353	70
gp47	tail fibre protein *gp47*	317/318	JBD30 tail fibre protein *gp48*	305/307	1.777	38
gp49	distal tail protein domain III	111/567	JBD30 distal tail protein domain IV	120/567	1.892	85
gp50	upper baseplate protein domain I	127/273	bacteriophage T5 baseplate hub protein HDI	158/949	0.896	34
gp53	baseplate hub protein domain II	123/735	bacteriophage T5 baseplate hub protein HDII	132/949	1.844	28
gp53	baseplate hub protein domain III	69/735	bacteriophage T5 baseplate hub protein HDIII	72/949	1.034	81
gp53	baseplate hub protein domain IV	78/735	bacteriophage T5 baseplate hub protein HDIV	64/949	1.106	77
gp53	baseplate hub protein domain V	116/735	bacteriophage T5 baseplate hub protein FNIII-1	92/949	2.782	77
gp53	baseplate hub protein domain VII	125/735	bacteriophage T5 baseplate hub protein FNIII-2	113/949	1.864	80
gp54	receptor binding protein thigh domain	117/382	bacteriophage vB_ApiP_P1 tail spike PDB-6e1r	554/554	3.221	15
gp54	receptor binding protein thigh domain	117/382	bacteriophage CBA120 tail spike PDB-6nw9	633/633	3.749	10
gp54	receptor binding protein thigh domain	117/382	bacteriophage T7 tail fibre PDB-7ey9	553/553	3.348	16

*
